# Down-regulation of circ0001361 induces apoptosis and suppresses the progression of glioma

**DOI:** 10.1371/journal.pone.0343681

**Published:** 2026-04-15

**Authors:** Xiaoqiong Zou, Ping Wang, Weixia Nong, Chang Liu, Feng Li, Chunhong Xue, Xin Li, Yanjing Wang, Yingying Ge, Qingmei Zhang, Bin Luo, Xiaoxun Xie

**Affiliations:** 1 Department of Histology and Embryology, School of Basic Medicine Science, Guangxi Medical University, Nanning, China; 2 Department of Neurosurgery, The First Affiliated Hospital of Guangxi Medical University, Nanning, China; 3 Key Laboratory of Preclinical Medicine (Guangxi Medical University), Education Department of Guangxi Zhuang Autonomous Region, Nanning, China; 4 Guangxi Higher Education Engineering Center of Advanced Technologies in Medical and Biological Intelligent Manufacturing, Nanning, China; 5 Key Laboratory of Early Prevention and Treatment of Regional High Frequency Tumor, Guangxi Medical University, Ministry of Education, Nanning, China; University of Navarra, SPAIN

## Abstract

**Background:**

Circ0001361 is a novel circRNA identified by our previous high-throughput sequencing of glioma. Here we explored the functional involvement of circ0001361 in gliomagenesis and elucidated its potential molecular mechanisms.

**Methods:**

The expression of circ0001361 in glioma was determined by qRT-PCR. CCK-8, colony formation assay, wound-healing, transwell assay, flow cytometric analysis and western blot were conducted to investigate cell proliferation, migration, invasion and apoptosis. The potential target miRNAs of circ0001361 and their downstream mRNAs were predicted by bioinformatics analysis and validated using dual-luciferase reporter assays.

**Results:**

Elevated expression of circ0001361 were observed in glioma tissues. The expression of circ0001361 was positively correlated with WHO tumor grades and Ki67 index, a well-established proliferation biomarker. Functional assays demonstrated that circ0001361 depletion inhibited cell proliferation, migration and invasion, while it promoted apoptosis. The bioinformatics analysis revealed that circ0001361 might target hsa-miR-525-5p, and further indicated that MEIS1 was a predicted downstream target of hsa-miR-525-5p. Subsequently, these predicted targeting relationships were both confirmed by dual-luciferase reporter assays.

**Conclusion:**

Circ0001361 enhances tumorigenic properties. Mechanistically, circ0001361 may regulate glioma progression via hsa-miR-525-5p/MEIS1, suggesting its potential as a therapeutic target for glioma intervention strategies.

## Background

Gliomas are formed by abnormal proliferation of glial cells that affect the brain and spinal cord [1,2]. The World Health Organization (WHO) categorizes gliomas into four grades (I-IV), with grades I – II representing low-grade gliomas (LGG), while grades III – IV are high-grade gliomas (HGG). The current standard therapy interventions, including radiotherapy, surgery, and temozolomide (TMZ) drug therapy, tend to lead to poor prognosis [3]. It may be due to the rapid proliferation, strong migration, and high aggressiveness of gliomas [4,5]. Therefore, exploring the molecular mechanisms, especially those associated with proliferation and apoptosis, and identifying novel molecular targets are of importance for clinical diagnosis and intervention.

Distinguished by their closed-loop conformation, circRNAs are a special class of RNA molecules that maintain structural continuity through covalent bonding, presenting neither 5′ terminal caps nor 3′ polyadenylated tails characteristic of linear RNA molecules [6,7]. CircRNAs are expressed conservatively in eukaryotic cells, which possess multiple pathophysiological functions [8]. Reports have demonstrated that circRNAs are actively involved in various cellular mechanisms, such as transcription, protein synthesis, and RNA degradation [9]. CircRNAs are promising clinical indicators for evaluating embryonic development, diseases progression and patients prognosis in various pathological conditions [10]. In recent years, circRNAs were reported to affect glioma progression. Therefore, it is of great significance for glioma’s targeted therapy to screen a novel circRNA and explore its role on glioma’s occurrence and development.

Our previous high-throughput sequencing [11] screened several upregulated circRNAs in glioma, including circ0001361. Circ0001361 is a novel RNA which was identified in lung adenocarcinoma, bladder cancer, liver cancer, neuroblastoma and breast cancer [12–17]. According to reports, circ0001361 was upregulated and facilitated the tumorigenesis and development of lung adenocarcinoma [12], and it was highly expressed in bladder cancer and promoted metastasis of tumor cells [13]. Additionally, circ0001361 was upregulated in liver cancer [14]. Circ0001361 regulated the viability and apoptosis of Neuroblastoma Cells [15]. Up-regulation of circ0001361 could alleviate axillary response after neoadjuvant chemotherapy in breast cancer [17]. However, there was no literature reported on circ0001361 in glioma. Therefore, we first detected circ0001361 expression in glioma, carried out malignant biological behavior experiments to verify its effect on glioma, and then conducted a preliminary investigation into its underlying molecular mechanisms.

## Methods

### Patients and tissue specimens

Glioma tissues were derived from 40 surgical patients (18 LGG and 22 HGG) at the First Affiliated Hospital of Guangxi Medical University. Inclusion criteria were as follows: postoperative pathological diagnosis of glioma, no prior radiotherapy or chemotherapy, and availability of complete clinical and follow-up data. Exclusion criteria were as follows: postoperative instability of vital signs, coexistence of another primary malignant tumor, and the presence of severe organic diseases or coagulation disorders. Additionally, control samples consisted of 10 histologically normal brain tissues resected during surgical approach procedures. General clinical data and pathological results of all patients were collected through the hospital medical record system. The data were accessed for research purposes in 12/8/2022. The recruitment period for this retrospective study, defined as the period during which patient cases were identified and their archived specimens were selected for analysis, was from 10/03/2022 to 01/06/2023. For data linkage and validation, authorized researchers temporarily accessed identifiable information under strict confidentiality protocols: all data were assigned unique study codes and stored in password-protected files. All analyses were performed on a final, de-identified dataset. The study protocol received ethical approval from the Ethics Committee of the First Affiliated Hospital of Guangxi Medical University (No. 20220098), with written informed consent obtained from all participants or their legal guardians.

### RNA sequencing

5 brain tissues and 5 glioma tissues were collected for high-throughput sequencing. The transcriptome sequencing data (RNA-Seq) comparing glioma cells to normal controls have been deposited in the Gene Expression Omnibus (GEO) under accession number GSE217950.

### Cell lines and cultivation

Glioma cells (SF763, SF126, U87, U251, A172) were maintained in DMEM medium containing 10% FBS, under standard culture conditions.

### RNA extraction and Quantitative real-time polymerase chain reaction (qRT‑PCR)

RNA was extracted using TRIzol reagent and reverse transcription was performed to obtained cDNA. QRT-PCR was conducted using ChamQ Universal SYBR qPCR Master Mix (Vazyme, China). Relative gene expression was analyzed using the 2^−ΔΔCt^. The specific primer sequences employed for this investigation have been documented in S1 Table.

### Sanger sequencing

The back-splice junction of circ0001361 was experimentally validated by reverse transcription-PCR followed by TA cloning and Sanger sequencing. The resulting sequence has been deposited in the European Nucleotide Archive (ENA) under accession number ERZ28559031.

### RNase R treatment

Linear RNA digestion was performed using RNase R (Geneseeed, China), with RNA samples being allocated into two experimental groups: an RNase R-treated group and an untreated control group. Both groups were placed at 37 °C for 25 minutes with 3 U/μg RNase R, followed by enzyme inactivation at 70 °C for 20 minutes.

### Cell transfection

Circ0001361 interference fragments si-circ0001361–1, si-circ0001361–2 and si-circ0001361–3 and negative control (NC) were synthesized by Sangon Bioteech. Then three interference sequences were transfected respectively using Lipofectamine 3000 reagent to downregulate the expression of circ0001361 in glioma cells. The transfection efficiency was verified by qPCR. The siRNA sequences are listed in S2 Table.

### Cell counting Kit-8 (CCK-8)

Glioma cells (3 × 10^3^) were plated in 96-well culture plates and maintained for varying durations (0, 24, 48, 72, and 96 h). Each well was supplemented with CCK-8 reagent for an additional 2 h incubation. The 450 nm absorbance readings were ultimately acquired with a microplate spectrophotometer.

### Colony formation assay

Cells (2 × 10^3^) were incubated in 6-well plates for 14 days, and processed with 4% paraformaldehyde for fixation, following by coloration with 0.1% crystal violet.

### Wound- healing assay

Glioma cells were distributed in 6-well plates, and subjected to scratching using a 200 µl pipette tip, followed by incubation with medium containing 2% FBS. The scratch area was finally observed and analyzed by image J.

### Transwell migration and invasion assay

Serum-starved cells were loaded into upper chambers, while lower chambers were supplemented with 20% FBS-supplemented medium. For the invasion assay, the upper chambers were pre-coated with Matrigel. Cells were observed with a microscope and counted by image J after 24 h. Matrigel was not used in the migration assay.

### Flow cytometric analysis

Cells were detected with an Annexin V-FITC/PI Apoptosis Detection Kit (Vazyme, China), while cell cycle was observed using a Cell Cycle Staining Kit (Multi Sciences, China). Flow cytometric analysis was performed for analysis.

### Western blot

Protein samples were electrophoresed on 10% SDS-PAGE gels and transfer to PVDF membranes. The membranes were subjected to blocking with 5% skim milk and following by primary antibodies, caspase-3, cleaved caspase-3 (CST #14220), caspase 9, cleaved caspase-9 (CST #9508) and GAPDH (CST #2118). Target proteins were ultimately detected via ECL, and images were finally acquired using an imaging analysis system.

### Dual-luciferase reporter assay

To validate the predicted interactions of circRNA-miRNA-mRNA, the wild-type (WT) or mutant (MUT) fragments of circ0001361 or MEIS1 3′UTR containing putative binding sites for hsa-miR-525-5p were synthesized (GenePharma, China). Cells were seeded in 24-well plate, and WT and MUT plasmids were co-transfected with miRNA NC or mimics. After 48 hours, the Firefly luciferase and Renilla Luciferase were detected using the Dual Luciferase Reporter Assay Kit (Vazyme, China). Firefly luciferase was normalized to Renilla Luciferase.

### Bioinformatics analysis

Circ0001361 was identified by circBase (http://www.circbase.org/). CircBank (http://www.circbank.cn/searchMiRNA.html) and ENCORI (http://starbase.sysu.edu.cn/index.php) were used to predict miRNAs targeted by circ0001361. mirDIP (http://ophid.utoronto.ca/mirDIP/), miRWalk (http://mirwalk.umm.uni-heidelberg.de/) and LinkedOmics (http://linkedomics.org/login.php) were used to predict target genes for miRNA. CGGA (https://www.cgga.org.cn/), GEPIA2 (http://gepia2.cancer-pku.cn/#index) and dbDEMC (https://www.biosino.org/dbDEMC/experiment/detail/EXP00539) were used to predict the expression and prognostic significance of miRNAs and their target genes in glioma.

### Statistical analysis

SPSS 26.0 software was conducted to analyze statistics and GraphPad Prism 5 was used to create charts. Quantitative results from triplicate independent experiments are expressed as means with standard deviation (mean ± SD). Results demonstrating statistical significance were identified by *P* < 0.05.

## Results

### Identification and characteristics of circ0001361 in glioma

**Fig 1:**
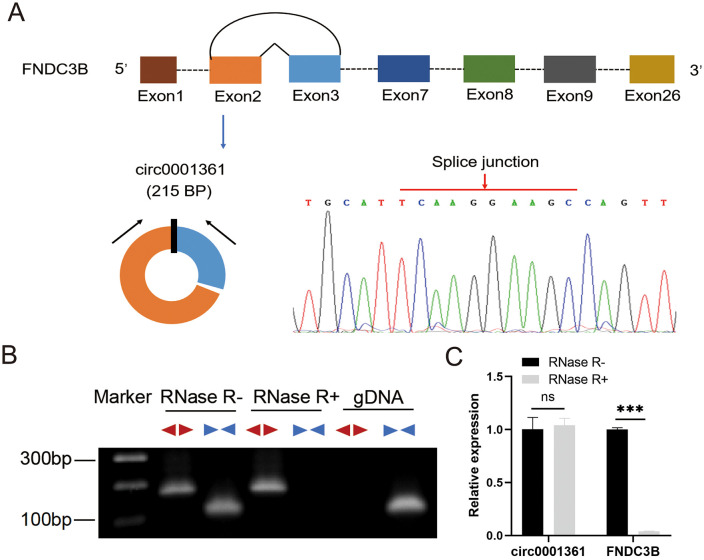
Identification and verification of circRNA. (A) Schematic illustration showed the FNDC3B exons 2–3 circularization to form circ0001361. The presence of circ0001361 was verified by Sanger sequencing, and the red arrow indicated the splice junction. (B) Agarose gel electrophoresis was used to detect circ0001361 and FNDC3B. The pattern 
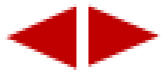
 represented circ0001361 and 
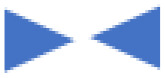
 represented FNDC3B. (C) RNase R assay was used to evaluate the stability of circ0001361 and FNDC3B in SF126. * *P* < 0.05, **P < 0.01, ***P < 0.001, ns means no significance.

In the high-throughput sequencing, differentially expressed circRNAs between glioma tissues and normal brain tissues were screened, in which circ0001361 was the 14th most up-regulated circRNA in glioma (Table 1). Circ0001361 was 215 bp in length and spliced by 2–3 exons of the Homo sapiens fibronectin type III domain containing 3B (FNDC3B) gene (GenBank: NM_022763), which was confirmed by Sanger sequencing (Fig 1A). However, we cannot exclude that head-tail splicing may be formed by genomic rearrangements. To exclude this possibility, special divergent primers and convergent primers were designed to amplify circ0001361 and FNDC3B mRNA, using cDNA and genomic DNA (gDNA) extracted from SF126 cells as templates. The findings demonstrated that circ0001361 was exclusively amplified from cDNA samples, with no detectable amplification from gDNA (Fig 1B). Subsequently, an RNase R digestion experiment was conducted, which confirmed the circular structure of circ0001361. In the result, RNase R could digest linear FNDC3B mRNA but not circ0001361, indicating that circ0001361 was more stable (Fig 1B, 1C).

**Table 1:** Top 15 up-regulated circRNAs in glioma tissues.

circRNA	Gene Name	log2FC	*p* value	q value
hsa_circ_0004658	EMILIN2	6.963494203	2.52258E-05	0.001338908
hsa_circ_0095448	SOX6	6.060787834	8.38401E-05	0.002856775
chr19:6697354-6697805:-	C3	5.798706026	0.000367238	0.005825151
hsa_circ_0114779	SLC24A3	5.567974179	0.000922468	0.010594263
hsa_circ_0021350	SOX6	5.395433017	1.50795E-05	0.000924878
hsa_circ_0079817	BBS9	5.374444025	0.002224703	0.018834912
hsa_circ_0006357	EZH2	5.362565143	0.002793626	0.022413974
hsa_circ_0000669	CARHSP1	5.263193293	0.003490054	0.026463044
hsa_circ_0001721	CDK14	5.24459019	0.002970592	0.023705958
hsa_circ_0001460	NEIL3	5.203160408	3.52111E-06	0.000404928
hsa_circ_0004490	UIMC1	5.175619173	0.004556558	0.032837736
hsa_circ_0099633	RMST	5.173048902	0.006931137	0.044385007
hsa_circ_0070253	SEC31A	5.142530479	0.004905527	0.03455761
**hsa_circ_0001361**	**FNDC3B**	**5.13100422**	**0.004759788**	**0.033858287**
hsa_circ_0004797	CNIH3	5.110695106	0.006164874	0.041000123

### Association between circ0001361 and clinicopathological characteristics in glioma patients

**Fig 2:**
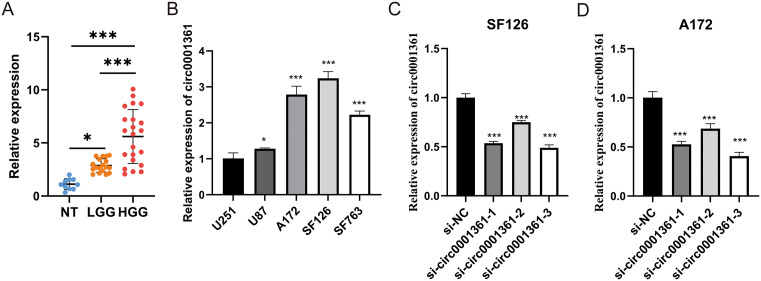
Circ0001361 expression in glioma and selection of its interfering sequences. (A) Circ0001361 was detected by qRT-PCR in normal brain (NT), low-grade glioma (LGG) and high-grade glioma (HGG). (B) Circ0001361 was detected by qRT-PCR in glioma cells and U251 was used as a control. (C&D) The interference efficiency of three interfering sequences was verified in SF126 and A172.

To further characterize clinicopathological significance of circ0001361 in glioma, expression of circ0001361 in 50 clinic tissue samples was detected, including 18 low-grade gliomas, 22 high-grade gliomas, and 10 normal tissues. It showed that circ0001361 expression was higher in glioma than in normal tissues. Subsequent investigations revealed elevated levels of circ0001361 in high-grade glioma specimens relative to the low-grade counterparts (Fig 2A). For evaluating the association of circ0001361 expression with glioma clinicopathological characteristics, a cohort of 40 glioma cases was stratified into two subgroups based on circ0001361 expression, using the median value in tumor tissues as the cutoff point (Table 2). We further investigate association between circ0001361 expression levels and clinicopathological features, including gender, age, glioma grade, tumor size, Karnofsky (KPS) and some immunohistochemical markers such as Ki67, P53, EMA (epithelial-membrane-antigen), GFAP (glial fibrillary acidic protein), and MGMT (O6 -methylguanine-DNA-methyltransferase). High expression of circ0001361 was associated with glioma high-grade and ki67 index, but not with gender, age, tumor size, KPS and other immunohistochemical markers, such as P53, EMA, GFAP and MGMT.

**Table 2:** Relative circ0001361 expression and clinicopathological features in glioma patients.

			circ0001361 expression n (%)		
Variables	Group	Sum	Low	High	*P* value
Age					0.749
	≤40	17	9(45)	8(40)	
	>40	23	11(55)	12(60)	
Gender					0.752
	Male	21	10(50)	11(55)	
	Female	19	10(50)	9(45)	
WHO grade					**0.001**
	I-II	18	14(70)	4(20)	
	III-IV	22	6(30)	16(80)	
Tumor size					0.752
	<5.0 cm	19	10(50)	9(45)	
	≥5.0 cm	21	10(50)	11(55)	
Ki67					**0.025**
	<20%	23	15(75)	8(40)	
	≥20%	17	5(25)	12(60)	
P53					0.752
	-/+	21	10(50)	11(55)	
	++/+++	19	10(50)	9(45)	
EMA					0.723
	–	29	14(70)	15(75)	
	+	11	6(30)	5(25)	
GFAP					1
	–	3	1(5)	2(10)	
	+	37	19(95)	18(90)	
MGMT					0.507
	–	14	6(30)	8(40)	
	+	26	14(70)	12(60)	
KPS					0.185
	<70	14	5(25)	9(45)	
	≥70	26	15(75)	11(55)	

*P*: Chi-squared test

KPS: Karnofsky Performance Score, EMA: epithelial-membrane-antigen, GFAP: glial fibrillary acidic protein, MGMT: O-6-Methylguanine DNA Methyltransferase

### Establishment of circ0001361-knockdown cell model

Additionally, the expression of circ0001361 was detected in glioma cells (A172, U251, U87, SF126 and SF763), and the expression of circ0001361 in SF126 and A172 was relatively high. Given that circ0001361 expression was highest in SF126 and A172 cells (Fig 2B), we selected these two cell lines for loss-of-function studies to investigate its necessity in maintaining aggressive phenotypes.To this end, we established circ0001361-knockdown cell model in SF126 and A172 with siRNA. Three si-circ0001361 interference sequences (si-circ0001361–1, si-circ0001361–2, and si-circ0001361–3) were designed and validated. All three siRNAs effectively knocked down circ0001361, with si-circ0001361–3 displaying highest efficiency (Fig 2C, 2D). Consequently, si-circ0001361–3 was selected for subsequent experiments.

### Influence of circ0001361 on proliferation, cell cycle and apoptosis in glioma cells

**Fig 3:**
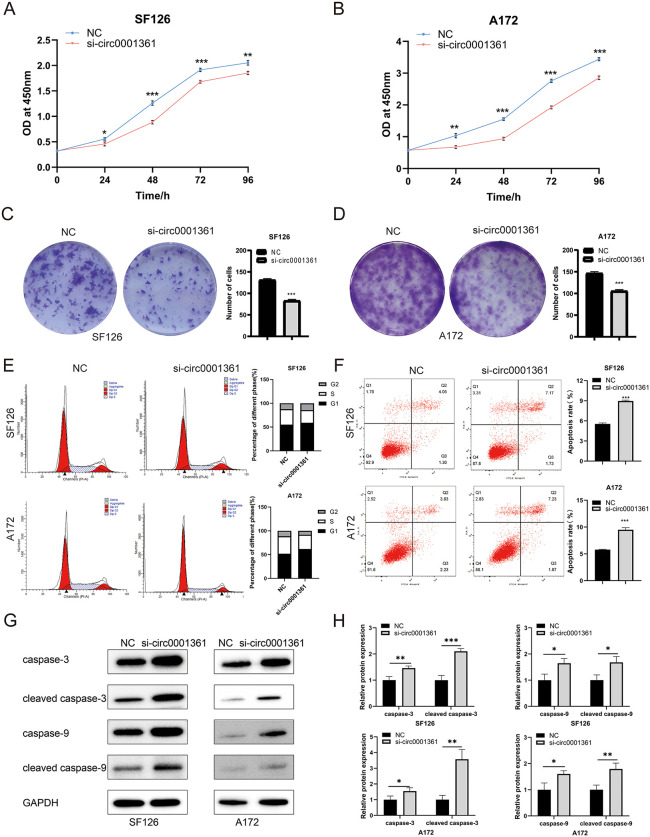
Proliferation inhibition, cell cycle arrest and apoptosis promotion by down-regulating circ0001361 in glioma cells. (A&B) Cell proliferation was evaluated by CCK-8 assay. (C&D) Colony formation assays were performed in glioma cells. (E) Cell cycle was monitored by flow cytometry. (F-H) Apoptosis rate was analyzed by flow cytometry and WB.

The CCK-8 assay revealed that knockdown of circ0001361 suppressed the growth of both glioma cell lines, but the time to achieve the strongest effect differed. In SF126 cells, the greatest growth inhibition occurred 48 hours post-transfection (Fig 3A). In contrast, A172 cells required 72 hours post-transfection to reach the maximum level of inhibition (Fig 3B). Colony formation showed that colony formation ability was reduced with circ0001361 down-regulation (Fig 3C, 3D). Together, these results revealed the involvement of circ0001361 in cell proliferation of glioma.

Cell cycle distribution was analyzed to quantify the percentage in G1, S, and G2 phases. A notable increase in G1 phase proportion was observed in the si-circ0001361 group, accompanied by a corresponding reduction in S phase distribution (Fig 3E). This indicated that the cell progression was inhibited by blocking in G1 phase after knocking down circ0001361.

Next, assessment of apoptosis showed that compared with NC group, cells transfected with si-circ0001361 could promote apoptosis (Fig 3F). Additionally, results of western blot (WB) found that expression of caspase-3, cleaved caspase-3, caspase-9 and cleaved caspase-9 was increased in si-circ0001361 group (Fig 3G, 3H). Therefore, it could be concluded that knocking down circ0001361 could enhance apoptosis of glioma cells.

### Enhancing effect of circ0001361 on the migration and invasion of glioma cells

**Fig 4:**
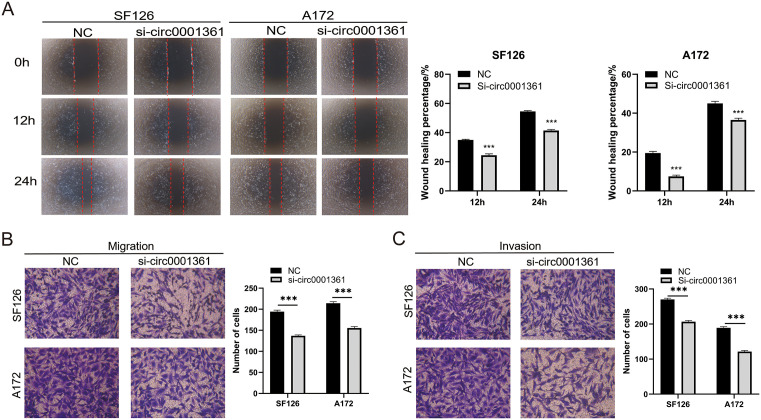
Suppression of glioma cell migration and invasion by down-regulation of circ0001361. (A&B) Migration capacity was detected by wound-healing essay and transwell migration assay. (C) Invasion capacity was evaluated by transwell invasion assays.

To further understand the association between the high expression of circ0001361 and high-grade glioma, wound-healing and transwell assays were conducted to determine whether circ0001361 promotes metastatic ability of glioma cells. Wound-healing assays demonstrated a marked reduction in cell migration following circ0001361 knockdown, with significantly lower healed areas in the knockdown group (54.6% vs. 41.4% in SF126 cells and 45.0% vs. 36.5% in A172 cells, respectively) (Fig 4A). These findings were corroborated by transwell assays, collectively indicating a diminished migratory potential in glioma cells (Fig 4B). Additionally, transwell invasion assay were conducted and demonstrated that invasion potential decreased after knocking down circ0001361 (Fig 4C).

Experimental data revealed that suppression of circ0001361 effectively attenuated migratory and invasive capacities of glioma cells.

### Identification of the circ0001361 – hsa-miR-525-5p - MEIS1 axis in glioma

**Fig 5:**
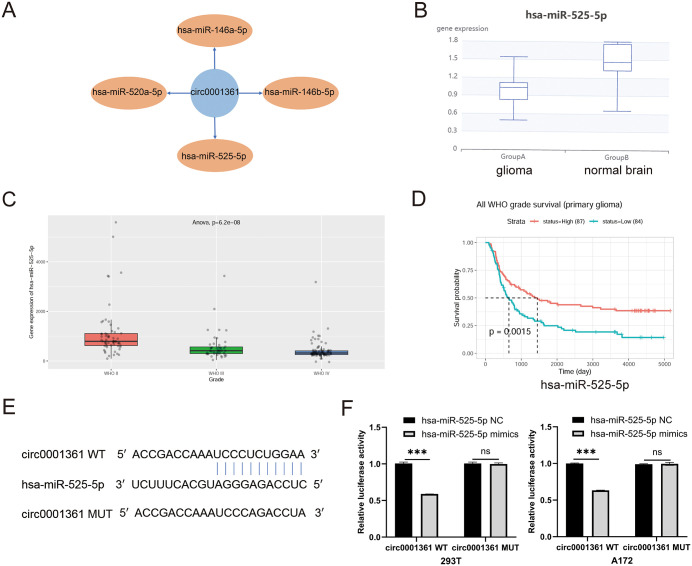
Identification of circ0001361 targeting hsa-miR-525-5p. (A) MiRNAs targeted by circ0001361 were predicted. (B) Expression of hsa-miR-525-5p in glioma (Group A) and normal brain tissue (Group B) was shown. (C) Expression of hsa-miR-525-5p in glioma grade Ⅱ -Ⅳ was shown. (D) Prognostic significance of hsa-miR-525-5p was analyzed. (E) Potential binding sites between circ0001361 and hsa-miR-525-5p were predicted. (F) Circ0001361-hsa-miR-525-5p interaction was confirmed by dual-luciferase reporter assay.

Based on the aforementioned carcinogenic role of circ0001361 in glioma, we preliminarily explored its molecular mechanism from the perspective of competing endogenous RNA (ceRNA) hypothesis. Potential miRNAs interacting with circ0001361 were predicted, leading to the selection of four candidates (hsa-miR-146a-5p, hsa-miR-146b-5p, hsa-miR-520a-5p and hsa-miR-525-5p) for downstream analysis. (Fig 5A). To identify key miRNAs, we first analyzed the expression and prognostic value of four candidate miRNAs in glioma using public databases. Initial screening revealed that hsa-miR-146a-5p and hsa-miR-146b-5p were upregulated in glioma, whereas hsa-miR-525-5p was downregulated compared with normal brain tissues (no expression data were available for hsa-miR-520a-5p in the databases). Furthermore, only hsa-miR-146b-5p and hsa-miR-525-5p exhibited a statistically significant association with patient overall survival (Fig 5B-5D, S1 Fig).

Based on this and supported by existing literature confirming hsa-miR-525-5 p’s interaction with circ0001361 in lung adenocarcinoma [12], we selected this miRNA as the most promising candidate for further investigation. Bioinformatic analysis demonstrated that low expression of hsa-miR-525-5p in glioma was associated with advanced tumor grade and worse patient survival (Fig 5C, 5D), suggesting its potential as an independent prognostic factor. As predicted by bioinformatic analysis (Fig 5E), potential binding sites exist between the circ0001361 and hsa-miR-525-5p. To validate this interaction, we performed a dual-luciferase reporter assay, which demonstrated a significant decrease in firefly luciferase activity for the wild-type reporter construct compared to its mutated counterpart, confirming a direct binding event (Fig 5F).

**Fig 6:**
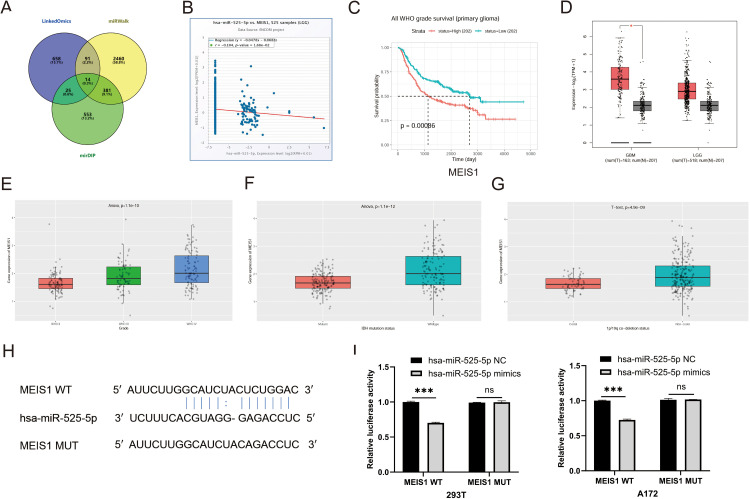
Identification of hsa-miR-525-5p targeting MEIS1. (A) Genes targeted by hsa-miR-525-5p were predicted. (B) Correlation between hsa-miR-525-5p and MEIS1 was analyzed. (C) Prognostic significance of MEIS1 was analyzed. (D) Expression of MEIS1 in GBM, LGG and normal brain tissues was shown. (E) Expression of MEIS1 in glioma grade Ⅱ -Ⅳ was shown. (F) The relationship between MEIS1 and IDH mutation status was shown. (G) The relationship between MEIS1 and 1p/19q co−deletion status was shown. (H) Potential binding sites between hsa-miR-525-5p and MEIS1 were predicted. (I) Hsa-miR-525-5p and MEIS1 interaction was confirmed by dual-luciferase reporter assay.

Following the preliminary verification of the circ0001361 – hsa-miR-525-5p interaction, we next sought to delineate the downstream target genes of this miRNA. Bioinformatics prediction initially yielded 14 potential target genes (Fig 6A). To prioritize candidates most consistent with the core ceRNA mechanism, we applied the principle of inverse expression correlation. Consequently, three genes (MEIS1, TMEM41B and VLDLR) showing a significant negative correlation with the miRNA’s expression level were selected as the most promising downstream targets for experimental validation (Fig 6B, S2 Fig). Survival analysis of the three candidate genes revealed that only MEIS1 expression exhibited a statistically significant association with patient overall survival, leading to its selection for in-depth analysis (Fig 6C, S3 Fig).

Analysis of public databases revealed that MEIS1 was highly expressed in glioma tissues (Fig. 6D). Furthermore, its expression level exhibited a positive correlation with tumor grade, with higher-grade gliomas displaying increased MEIS1 levels (Fig 6E). The expression of MEIS1 varied significantly across molecular subtypes of glioma. Specifically, its expression was significantly higher in IDH-wildtype tumors compared to IDH-mutant tumors (Fig 6F). Similarly, MEIS1 expression was markedly elevated in 1p/19q non-codel tumors relative to codel tumors (Fig 6G). The predicted miRNA binding sites on MEIS1, as illustrated in Fig 6H, were validated by a dual-luciferase reporter assay, which showed specific suppression of luciferase activity for the wild-type construct (Fig 6I).

## Discussion

Glioma refers to a type of tumors that originates in glial cells of brain. Because of the highly infiltration and invasion of vital brain regions, resection to obtain a negative tumor margin is nearly impossible [18]. Traditional treatments for gliomas were limited with poor prognosis and patients of gliomas have limited survival and high mortality rates [19]. The pathogenesis and progression of glioma involve multifaceted biological mechanisms and intricate molecular interactions, and the precise etiological factors underlying glioma genesis remain to be fully elucidated [20]. Therefore, elucidating of molecular mechanisms and seeking more effective therapies are urgently needed.

With swift progress of high-throughput sequencing, circRNAs have attracted much attention. Due to the closed circular structure of circRNA molecules, they are not affected by RNA exonuclease and are not easily degraded [21], which have longer half-life than linear RNAs [22,23]. Given the stability and conservation of circRNAs, they serve as biomarkers for multiple cancers, including glioma. According to a report, up-regulation of circ01844 induced apoptosis and suppressed proliferation of glioblastoma cells [24]. Circ0008344 promoted glioma progression and angiogenesis presumably [25]. It was also confirmed that exosome-mediated transfer of circGLIS3 enhanced temozolomide (TMZ) resistance in glioma cells [26]. These studies suggest the importance of circRNAs in the therapeutic landscape of gliomas.

In early high-throughput sequencing, we identified circ0001361 was up-regulated in glioma. The roles of circ0001361 have been reported in liver cancer [14], bladder cancer [13] and lung cancer [12], neuroblastoma [15,16] and breast cancer [17], relevant studies in glioma are still absent. Our investigation confirmed a significant elevation of circ0001361 expression in glioma specimens. Subsequent experiments in vitro showed that circ0001361 promoted cell migration, invasion and proliferation, and inhibited apoptosis. Further studies revealed a significant association between circ0001361 expression and glioma WHO grading. Furthermore, a significant positive correlation was observed between circ0001361 expression and the tumor proliferation rate, as assessed by the Ki67 index. Clinically, a high Ki67 index is a routine pathological parameter indicative of aggressive tumor behavior and is consistently linked to poorer prognosis in diverse malignancies [27,28]. Therefore, the positive correlation between circ0001361 and Ki67 index suggested its involvement in glioma proliferation, as subsequently confirmed by CCK-8 and colony formation assays. Together, our findings suggest that circ0001361 serves as a promising biomarker for assessing glioma malignancy and patient prognosis, holding dual promise for improving clinical diagnosis and informing future targeted therapies.

CircRNAs are known to play a key role in tumorigenesis primarily by competitively adsorbing miRNAs and functioning as miRNA sponges [29]. Based on this principle, circ0001361 is proposed to regulate the occurrence and progression of glioma through circRNA-miRNA-mRNA network. To explore the role of circ0001361, hsa-miR-525-5p was identified as the target of circ0001361 by both bioinformatic analysis and luciferase reporter assay. It has been reported that circ0001361 interacted with hsa-miR-525-5p in lung adenocarcinoma [12]. Here, our results further confirmed this interaction in glioma. A review of the literature indicates that hsa-miR-525-5p acts as a tumor suppressor in multiple cancer types, such as cervical cancer [30], ovarian cancer [31] and non-small cell lung cancer [32]. Our observation that elevated hsa-miR-525-5p expression correlates with improved prognosis in glioma implies a potential tumor-suppressive function in this malignancy, suggesting circ0001361 may promote the malignant phenotype of glioma by adsorbing hsa-miR-525-5p.

Our preliminary experiments suggest that circ0001361 may act as a “molecular sponge,” sequestering hsa-miR-525-5p and thereby relieving its inhibitory effect on downstream genes. Combining bioinformatic analysis and experimental validation, we confirmed that MEIS1 is a direct target of hsa-miR-525-5p. Our data analysis indicates that MEIS1 is a potential poor prognostic marker in glioma. Its high expression in tumor tissues correlates with shorter patient survival and is enriched in IDH-wildtype and 1p/19q non-codel gliomas. These findings collectively suggest that MEIS1 may drive the malignant progression of gliomas with specific molecular subtypes. MEIS1 functions as an essential HOX cofactor in organ development and physiological hematopoiesis. It plays an essential role in maintaining stemness, regulating transcription of self-renewal genes, and controlling developmental and differentiation programs, thereby functioning as an oncogenic driver in multiple tumor types [33,34]. Previous studies have established MEIS1 as a key oncogenic player in glioma. Its expression was regulated by risk enhancers and drives the transcription of downstream oncogenes such as SOX18, directly linking genetic variation in non‑coding regions to glioma progression [35]. Another study has demonstrated that MEIS1 is specifically upregulated in glioma stem cells, where its expression correlates with cell cycle genes, promotes tumor growth, and predicts poor prognosis, establishing it as a critical oncogenic factor and prognostic biomarker [36]. Together, these lines of evidence consolidate MEIS1 as a central regulator in glioma malignant progression. Building on this, our study further proposes that circ0001361 may relieve the inhibition on MEIS1 by sponging hsa-miR-525-5p, thereby forming a novel ceRNA regulatory axis (circ0001361/hsa-miR-525-5p/MEIS1). This axis thus offers a post‑transcriptional mechanistic insight into the aberrant overexpression of MEIS1 in glioma, while also highlighting a potential therapeutic vulnerability within its upstream regulatory network.

Collectively, our data suggest that circ0001361 may function via the hsa-miR-525-5p/MEIS1 axis in glioma, adsorbing the miRNA to upregulate this oncogenic target and ultimately driving aggressive phenotypes including proliferation and metastasis. Of course, this proposed mechanism awaits further validation through subsequent rescue experiments to be fully established.

## Conclusion

Elevated circ0001361 as a novel oncogenic factor that promotes glioma progression, with its function potentially linked to the hsa-miR-525-5p/MEIS1 pathway, highlighting its promise as a therapeutic target for glioma.

## Supporting information

S1 Fig Expression and survival analysis of hsa-miR-146a-5p, hsa-miR-146b-5p and hsa-miR-520a-5p in glioma. (A) Expression of hsa-miR-146a-5p and hsa-miR-146b-5p in glioma (Group A) and normal brain tissue (Group B) was shown. (B) Expression of hsa-miR-146a-5p, hsa-miR-146b-5p and hsa-miR-520a-5p in glioma grade Ⅱ -Ⅳ was shown. (C) Prognostic significance of hsa-miR-146a-5p, hsa-miR-146b-5p and hsa-miR-520a-5p was analyzed.
(TIF)

S2 Fig Correlations between hsa-miR-525-5p expression and various target genes in glioma cells. (A) FOXJ3, (B) BTF3L4, (C) DMD, (D) CELF2, (E) NPR3, (F) RNF220, (G) MTF1, (H) NR6A1, (I) WDR37, (J) PHACTR4, (K) ZNF518A, (L) TMEM41B, (M) VLDLR.
(PDF)

S3 Fig Expression and survival analysis of TMEM41B and VLDLR in glioma. (A) Correlation between hsa-miR-525-5p and TMEM41B and VLDLR was analyzed. (B) Expression of TMEM41B and VLDLR in GBM, LGG and normal brain tissues was shown. (C) Expression of TMEM41B and VLDLR in glioma grade Ⅱ -Ⅳ was shown. (D) Prognostic significance of TMEM41B and VLDLR was analyzed. (E) The relationship between TMEM41B, VLDLR and IDH mutation status was shown. (F) The relationship between TMEM41B, VLDLR and 1p/19q co−deletion status was shown.
(TIF)

S1 Table Primer sequences. (DOCX)

S2 Table SiRNA sequences. (DOCX)

S1 File Raw images. (PDF)
