# Supplementary figures and images for: Down-regulation of circ0001361 induces apoptosis and suppresses the progression of glioma
Source: PLoS One. 2026 Apr 15;21(4):e0343681. doi: 10.1371/journal.pone.0343681 (PMC13082647; doi:10.1371/journal.pone.0343681)

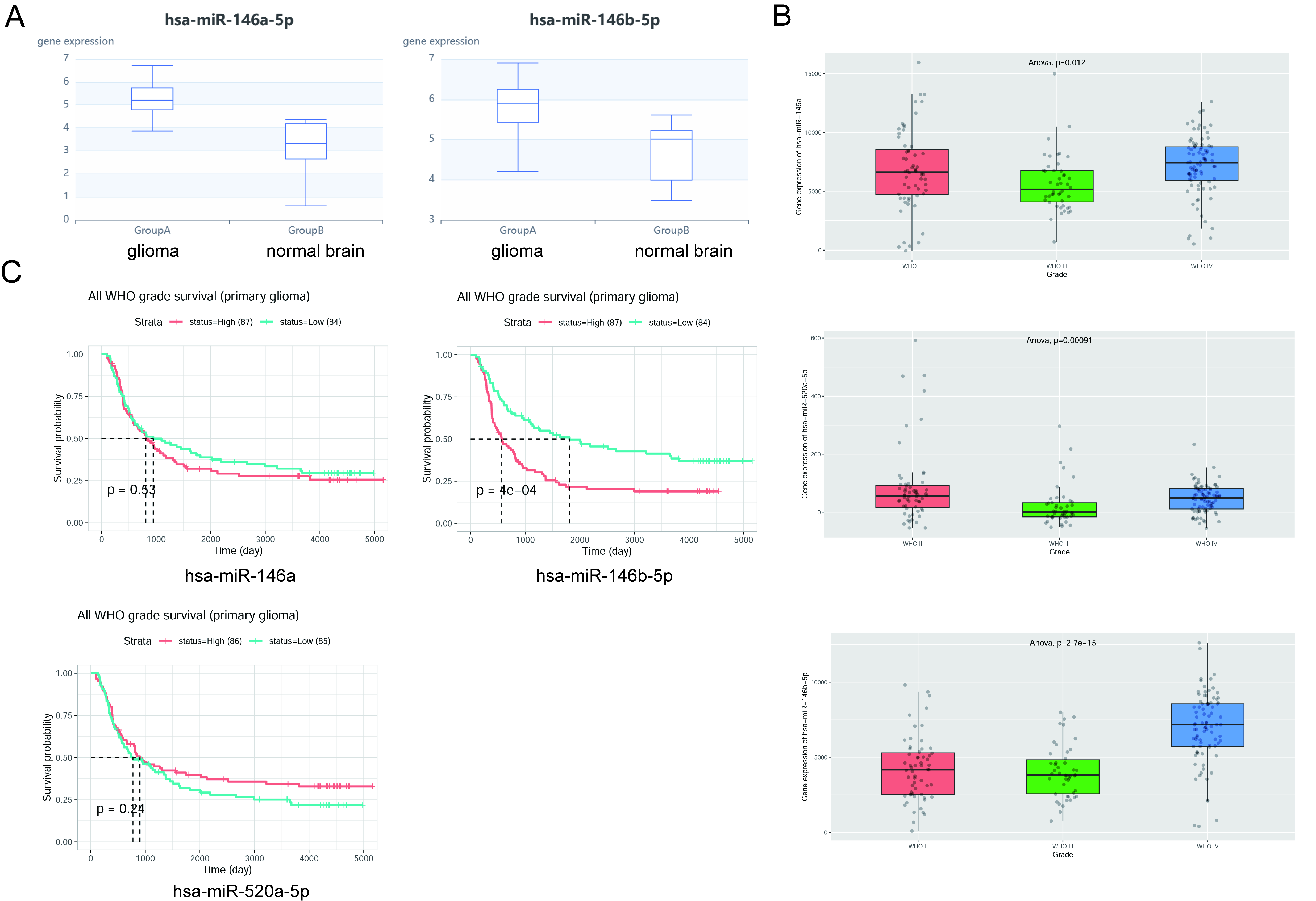

Supplement: S1 Fig — (A) Expression of hsa-miR-146a-5p and hsa-miR-146b-5p in glioma (Group A) and normal brain tissue (Group B) was shown. (B) Expression of hsa-miR-146a-5p, hsa-miR-146b-5p and hsa-miR-520a-5p in glioma grade Ⅱ -Ⅳ was shown. (C) Prognostic significance of hsa-miR-146a-5p, hsa-miR-146b-5p and hsa-miR-520a-5p was analyzed. (TIF) [file pone.0343681.s001.tif]

A

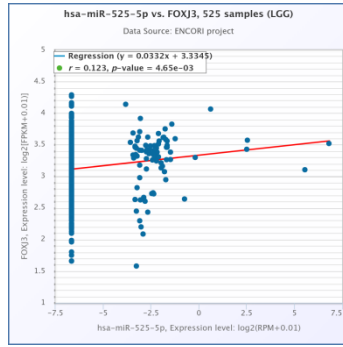

B

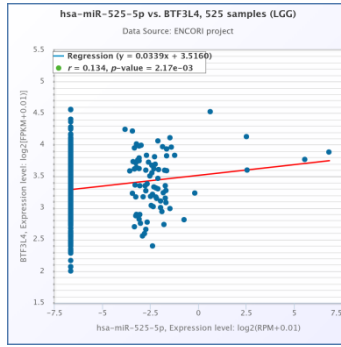

C

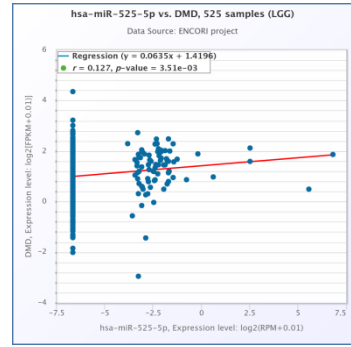

D

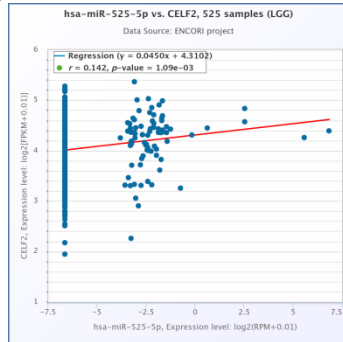

E

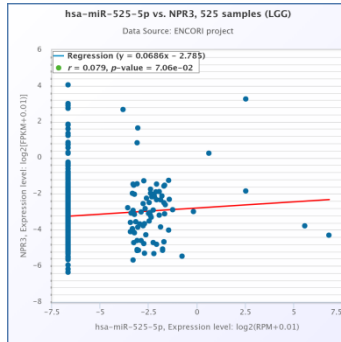

F

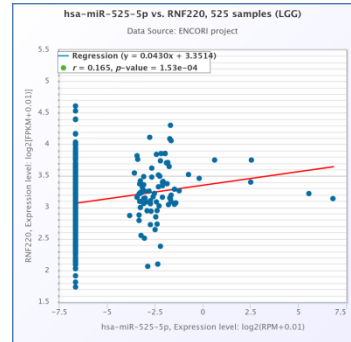

G

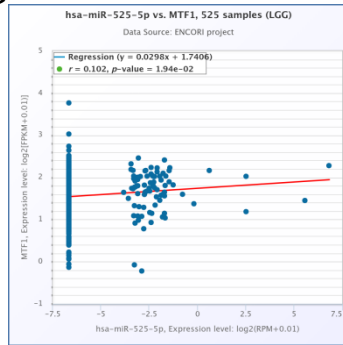

H

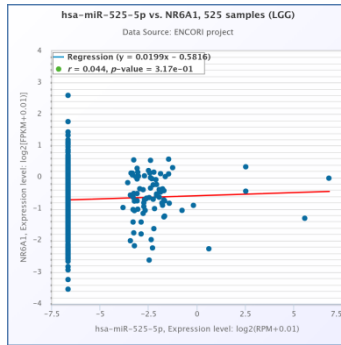

I

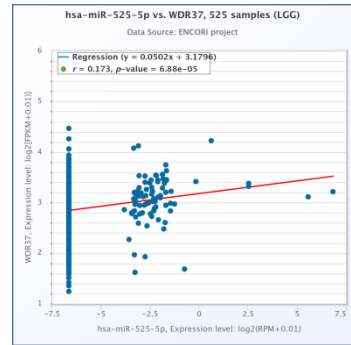

J

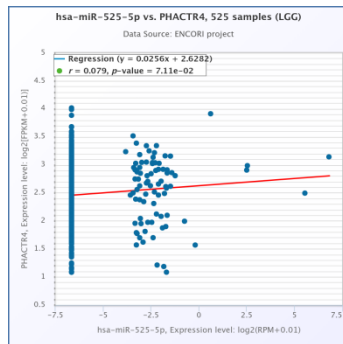

K

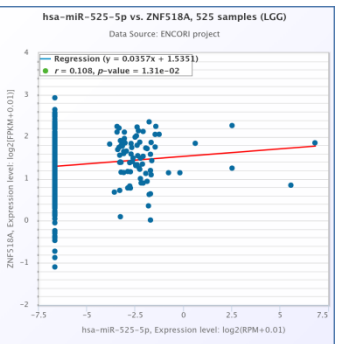

L

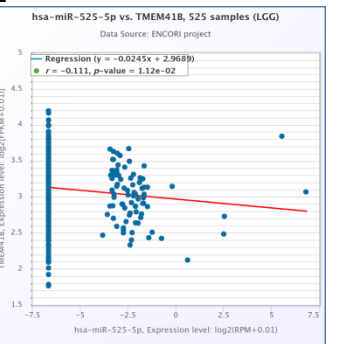

M

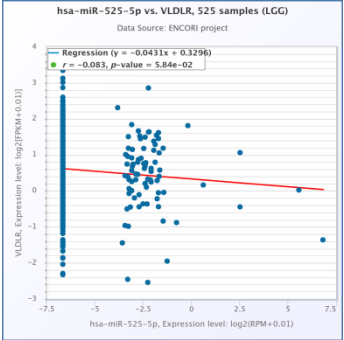

Supplement: S2 Fig — (A) FOXJ3, (B) BTF3L4, (C) DMD, (D) CELF2, (E) NPR3, (F) RNF220, (G) MTF1, (H) NR6A1, (I) WDR37, (J) PHACTR4, (K) ZNF518A, (L) TMEM41B, (M) VLDLR. (PDF) [file pone.0343681.s002.pdf]

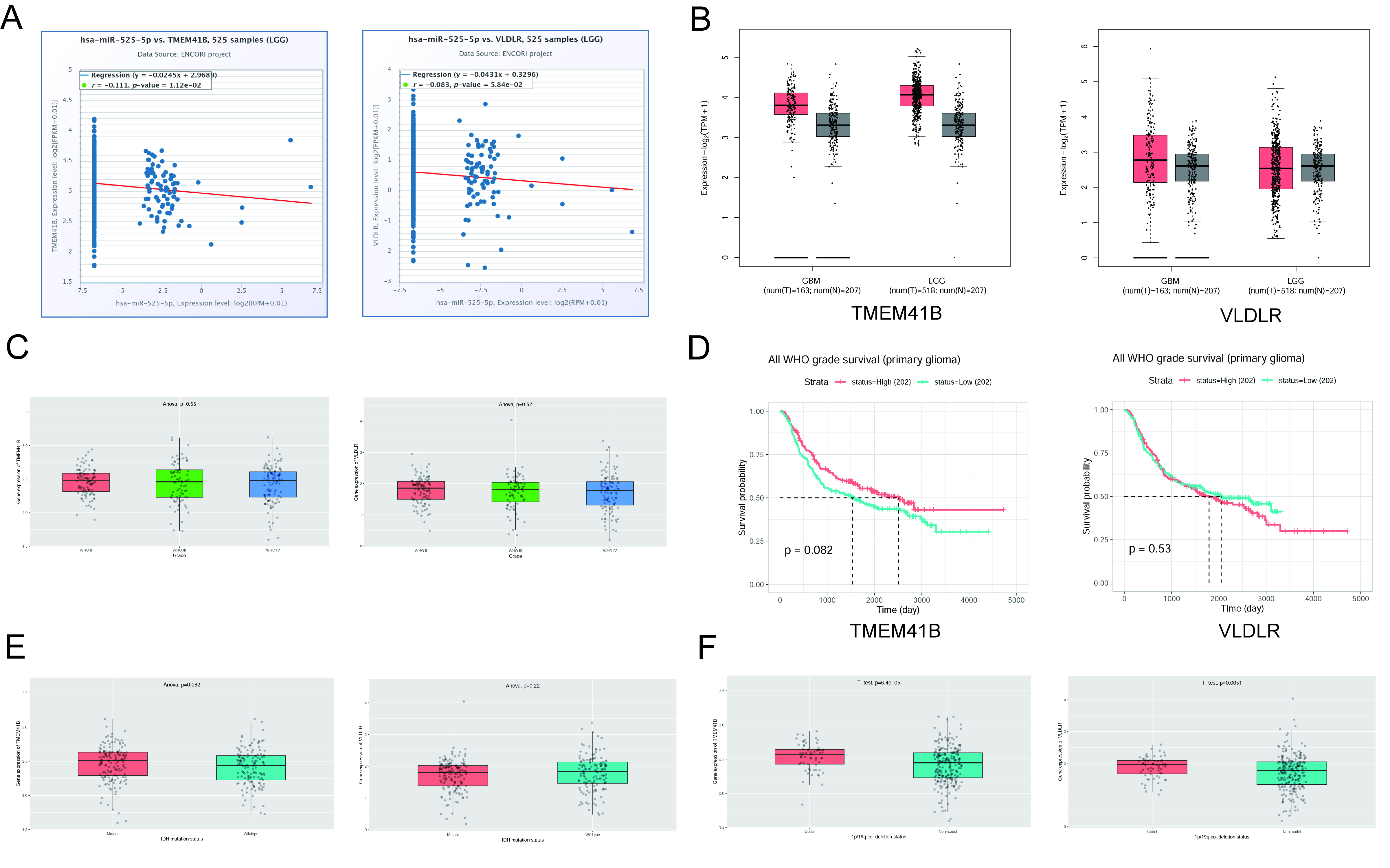

Supplement: S3 Fig — (A) Correlation between hsa-miR-525-5p and TMEM41B and VLDLR was analyzed. (B) Expression of TMEM41B and VLDLR in GBM, LGG and normal brain tissues was shown. (C) Expression of TMEM41B and VLDLR in glioma grade Ⅱ -Ⅳ was shown. (D) Prognostic significance of TMEM41B and VLDLR was analyzed. (E) The relationship between TMEM41B, VLDLR and IDH mutation status was shown. (F) The relationship between TMEM41B, VLDLR and 1p/19q co−deletion status was shown. (TIF) [file pone.0343681.s003.tif]
