# Supplementary material for: Down-regulation of circ0001361 induces apoptosis and suppresses the progression of glioma
Source: PLoS One. 2026 Apr 15;21(4):e0343681. doi: 10.1371/journal.pone.0343681 (PMC13082647; doi:10.1371/journal.pone.0343681)
Supplement: S1 Table — (DOCX) [file pone.0343681.s005.docx]

S1 Table. Primer sequences

| Primer | Sequence (5′-3′) | Product length (bp) |
| --- | --- | --- |
| GAPDH forward | GCACCGTCAAGGCTGAGAAC | 138 |
| GAPDH reverse | TGGTGAAGACGCCAGTGGA | 138 |
| Circ0001361 forward | TGCAGCTCAGCAGGTTATTCT | 188 |
| Circ0001361 reverse | GCTACCTCTCCGTTCAGCAA | 188 |
| FNDC3B forward | ACGTCACAATGATGATGACCGA | 101 |
| FNDC3B reverse | AGCTGCATCTCCATTCACCA | 101 |
