# Supplementary material for: Down-regulation of circ0001361 induces apoptosis and suppresses the progression of glioma
Source: PLoS One. 2026 Apr 15;21(4):e0343681. doi: 10.1371/journal.pone.0343681 (PMC13082647; doi:10.1371/journal.pone.0343681)
Supplement: S2 Table — (DOCX) [file pone.0343681.s006.docx]

S2 Table. SiRNA sequences

| Primer | Sequence (5′-3′) |
| --- | --- |
| si-circ0001361-1 sense | GUGCAUUCAAGGAAGCCAGUUTT |
| si-circ0001361-1 antisense | AACUGGCUUCCUUGAAUGCACTT |
| si-circ0001361-2 sense | GCAUUCAAGGAAGCCAGUUGATT |
| si-circ0001361-2 antisense | UCAACUGGCUUCCUUGAAUGCTT |
| si-circ0001361-3 sense | UCAGUGCAUUCAAGGAAGCCATT |
| si-circ0001361-3 antisense | UGGCUUCCUUGAAUGCACUGATT |
| NC sense | UUCUCCGAACGUGUCACGUTT |
| NC antisense | ACGUGACACGUUCGGAGAATT |
