# Supplementary material for: Down-regulation of circ0001361 induces apoptosis and suppresses the progression of glioma
Source: PLoS One. 2026 Apr 15;21(4):e0343681. doi: 10.1371/journal.pone.0343681 (PMC13082647; doi:10.1371/journal.pone.0343681)
Supplement: S1 File — (PDF) [file pone.0343681.s004.pdf]

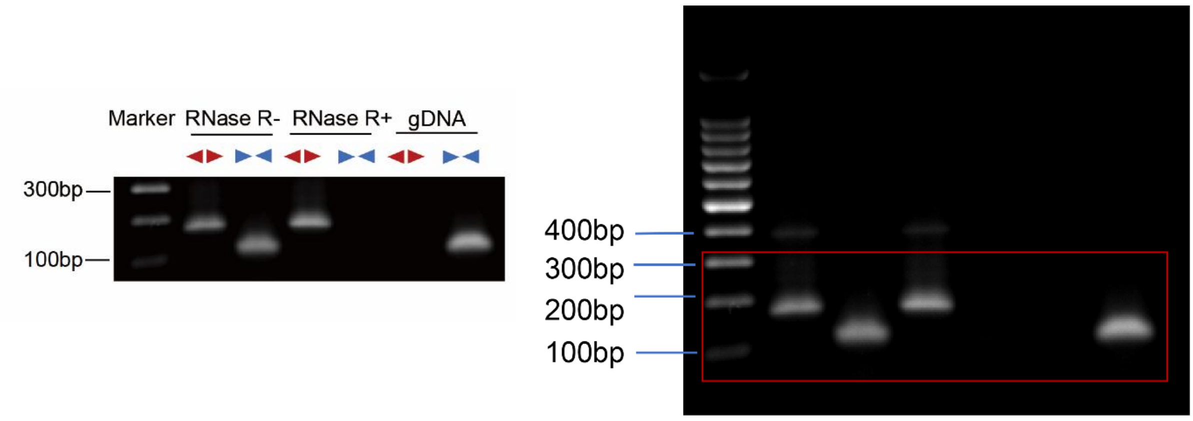

S1_Raw_Image A. Original agarose gel electrophoresis for Figure 1B

Note: The faint 400 bp band in raw data may be a primer dimer.

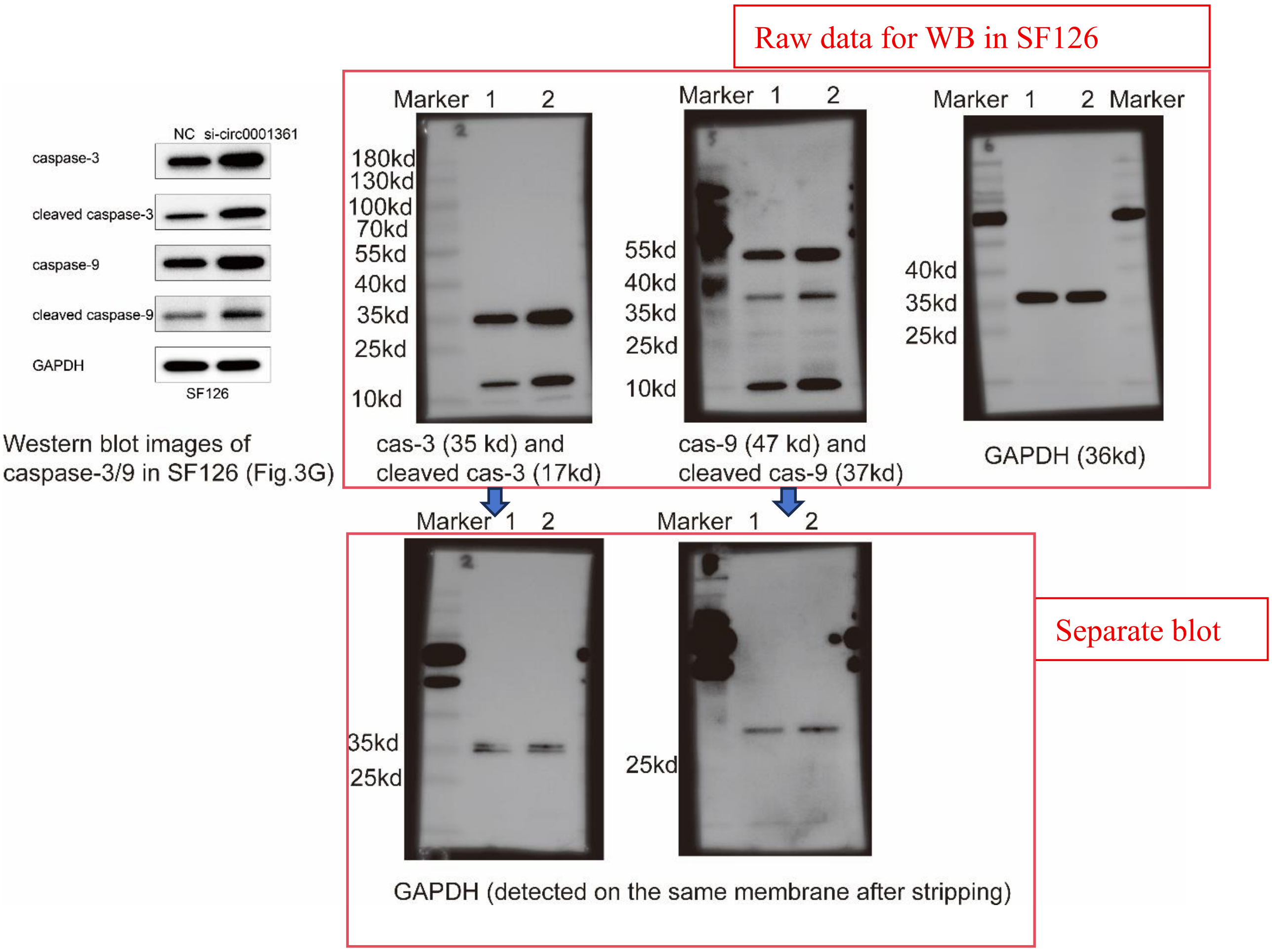

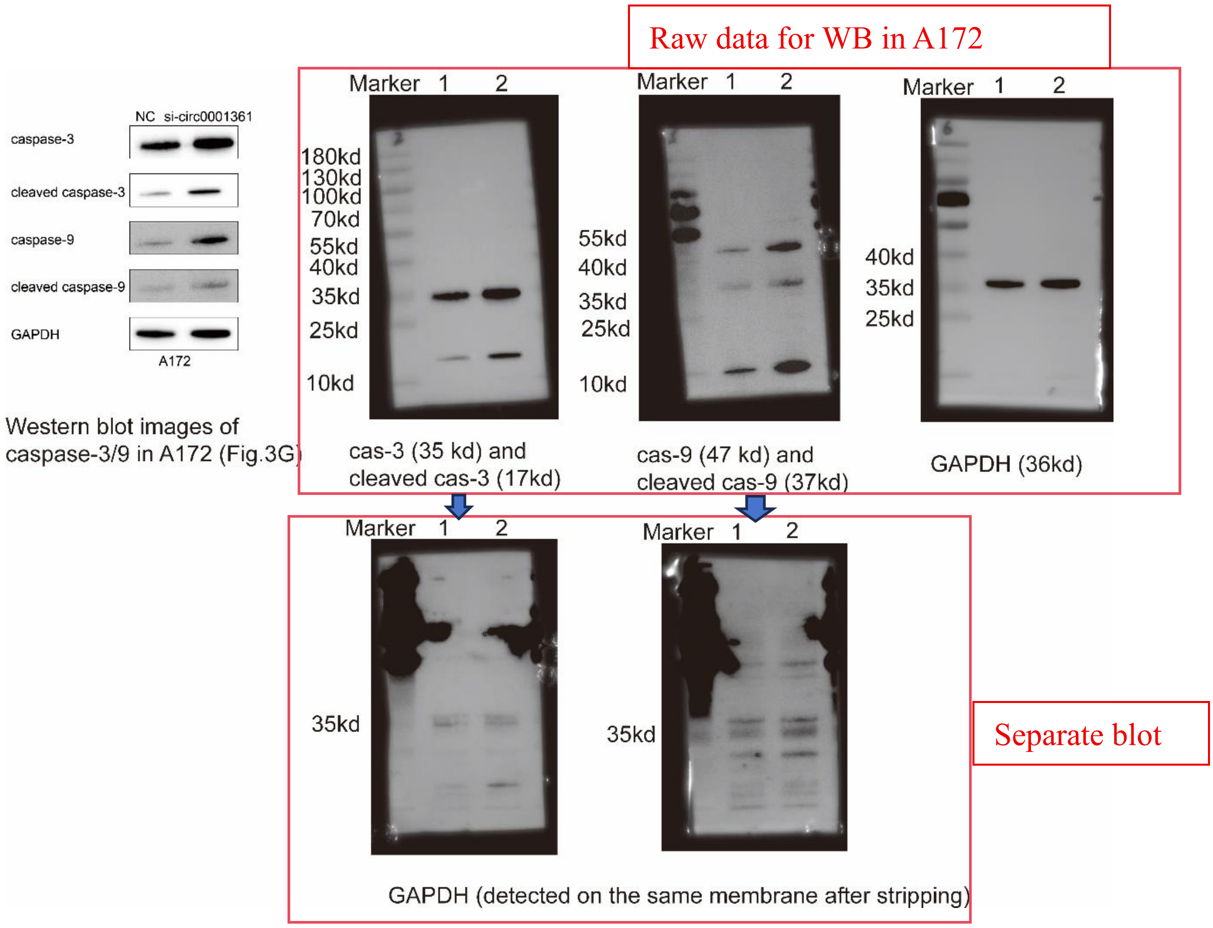

S1_Raw_Image B. Original western blot data for Figure 3G

Note:

(1) To confirm equal loading, we initially attempted to strip and re-probe the same membrane with an anti-GAPDH antibody. However, due to incomplete stripping that resulted in high residual background and compromised signal integrity (as shown in separate blot), we opted for the more reliable approach of running parallel gels for the target protein and GAPDH.

(2) In the detection of caspase-9/cleaved caspase-9, a non-specific band at approximately 10 kDa was observed, which is commonly attributed to the detection of IgG fragments from the blocking agent or serum by the secondary antibody. The band appears uniformly across all samples and is distinctly different in molecular weight from our target protein Caspase-9 (~47 kDa) and its active fragment (~35 kDa). Therefore, it does not affect any of our analysis or conclusions regarding the expression or cleavage of the target protein.

(3) After transfer, the PVDF membranes were numbered with a pencil for identification during subsequent antibody incubation steps.

(4) Lane 1: negative control (NC); Lane 2: si-circ0001361.

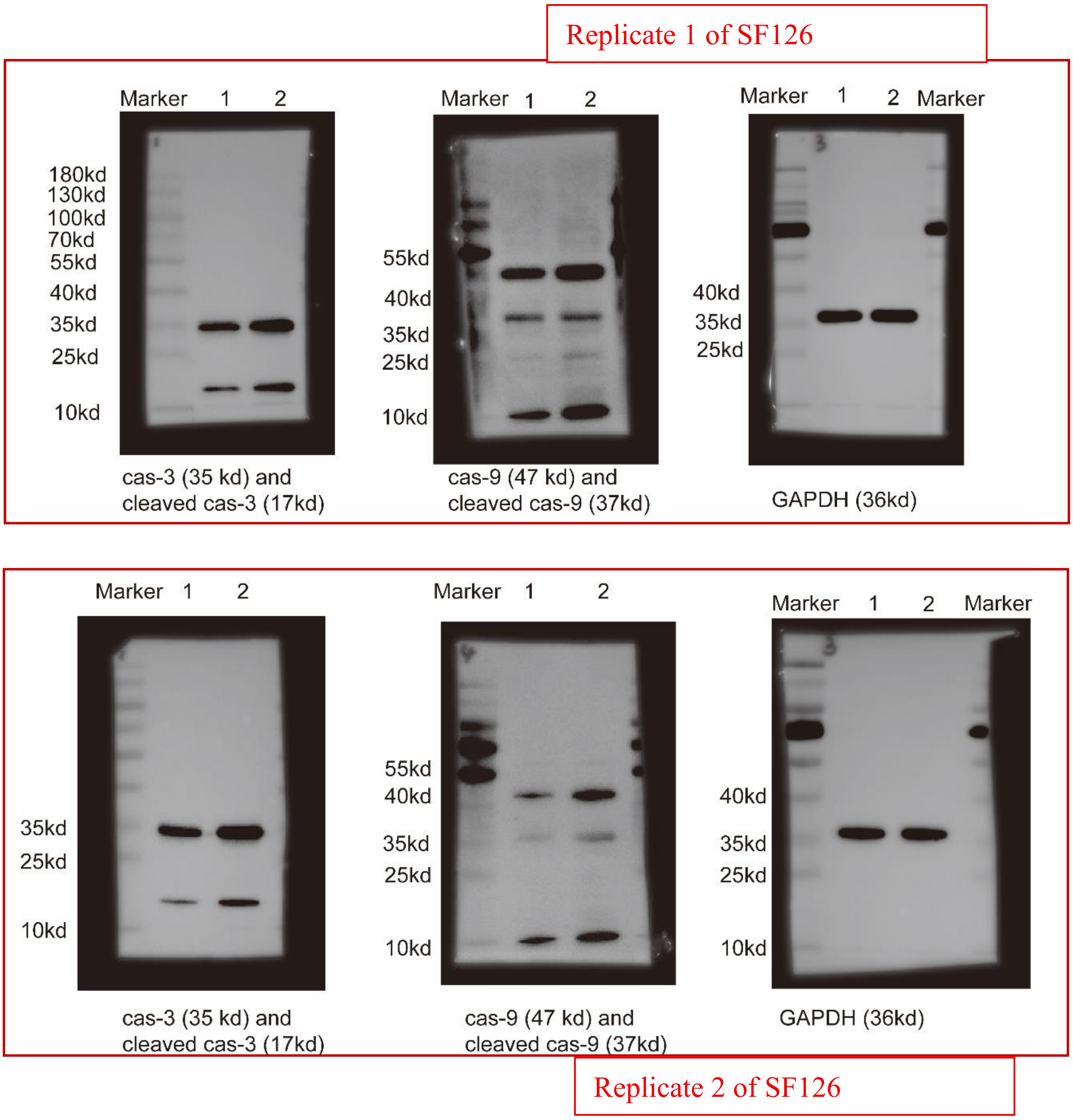

S1_Raw_Image C. Additional western blot data from two independent replicate experiments in SF126

Note: Lane 1: negative control (NC); Lane 2: si-circ0001361.

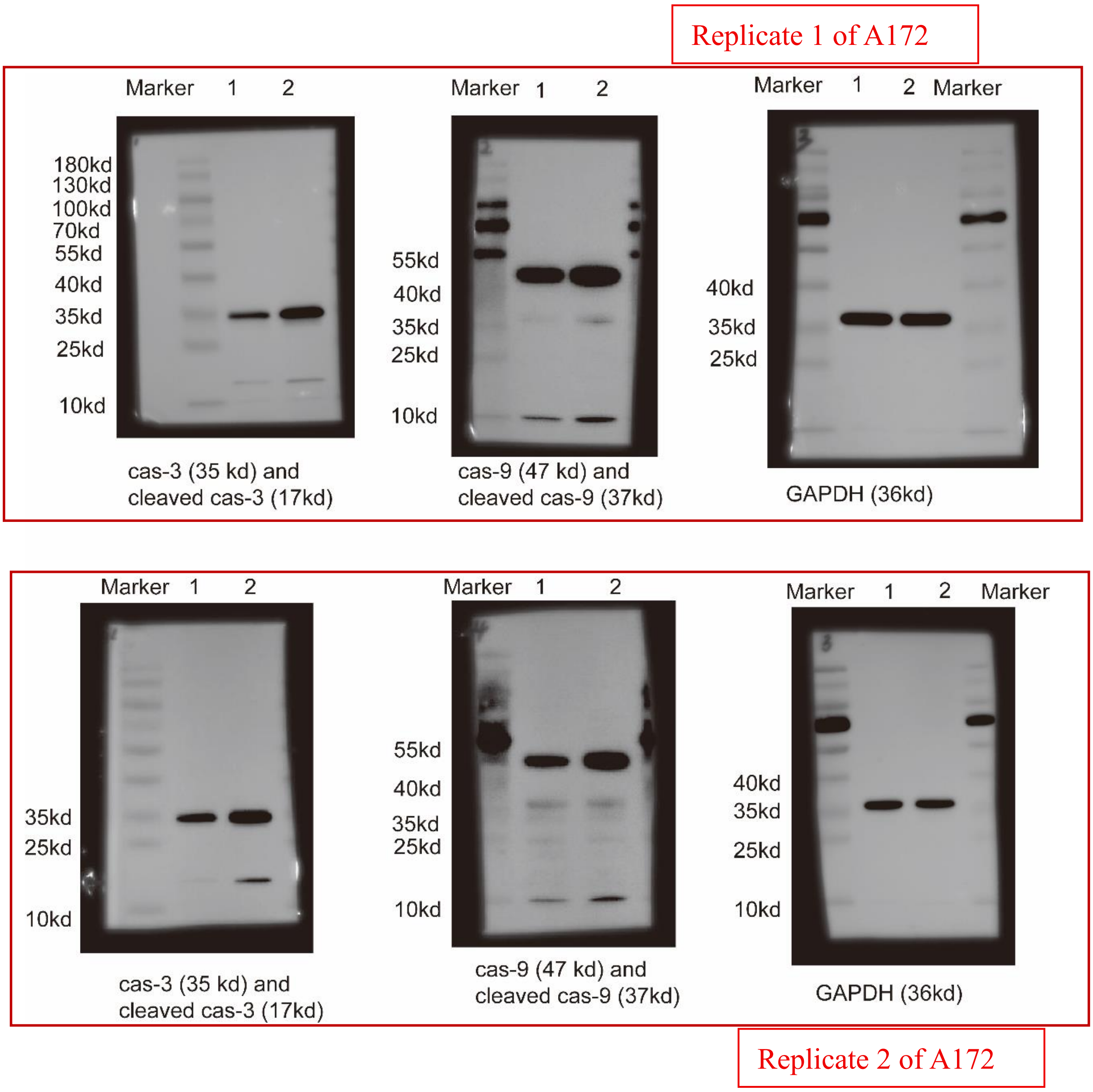

S1_Raw_Image D. Additional Western blot data from two independent replicate experiments in A172

Note: Lane 1: negative control (NC); Lane 2: si-circ0001361.
